# Antibiotic Resistance of Bacteria Isolated from Clinical Samples and Organs of Rescued Loggerhead Sea Turtles (*Caretta caretta*) in Southern Italy

**DOI:** 10.3390/ani14142103

**Published:** 2024-07-18

**Authors:** Emanuele Esposito, Antonino Pace, Andrea Affuso, Maria Oliviero, Doriana Iaccarino, Gianluigi Paduano, Fulvio Maffucci, Giovanna Fusco, Esterina De Carlo, Sandra Hochscheid, Fabio Di Nocera

**Affiliations:** 1 Istituto Zooprofilattico Sperimentale del Mezzogiorno, Via Salute 2, 80055 Portici, Italy; maria.oliviero@izsmportici.it (M.O.); doriana.iaccarino@izsmportici.it (D.I.); gianluigi.paduano@izsmportici.it (G.P.); giovanna.fusco@izsmportici.it (G.F.); direzionesanitaria@izsmportici.it (E.D.C.); fabio.dinocera@izsmportici.it (F.D.N.); 2 Department of Marine Animal Conservation and Public Engagement, Stazione Zoologica Anton Dohrn, Via Nuova Macello 16, 80055 Portici, Italy; andrea.affuso@szn.it (A.A.); fulvio.maffucci@szn.it (F.M.); sandra.hochscheid@szn.it (S.H.)

**Keywords:** wildlife, antimicrobials, multidrug resistance, one health, antimicrobial stewardship, sea turtles, *Caretta caretta*

## Abstract

**Simple Summary:**

Antimicrobial resistance is threatening health and the economy worldwide, not only in clinical settings, but also in livestock and wildlife management. Among the numerous wild animals affected, sea turtles are particularly exposed to antibiotics, due to biological and habitat characteristics. This study analysed the hospitalisation records in a sea turtle rescue centre in southern Italy during the last decade, focusing on the antibiotic resistance of bacteria isolated from clinical samples and organs. Resistance patterns to 7 antibiotics were examined in 138 bacteria isolated from 60 loggerhead sea turtles, highlighting 6 main bacterial families with different resistance rates. *Vibrionaceae* represented the predominant family, although the resistance rates did not exceed 10.5%. Similar results were described for *Shewanellaceae*, whereas the less represented families were those with the highest resistance rates and numbers of multidrug-resistant strains, especially *Enterobacteriaceae* and *Morganellaceae*. Previous antibiotic therapies appeared to enhance resistance, emphasizing the need to improve antimicrobial stewardship. Isolated bacteria are considered opportunistic pathogens, but their antibiotic resistance might compromise treatment and rehabilitation, potentially affecting the population health of these endangered species. Given the possible transfer of resistance across species, these findings should be considered from a One Health perspective, including other animals, humans and the environment.

**Abstract:**

Antimicrobial resistance affects all environments, endangering the health of numerous species, including wildlife. Increasing anthropic pressure promotes the acquisition and dissemination of antibiotic resistance by wild animals. Sea turtles, being particularly exposed, are considered sentinels and carriers of potential zoonotic pathogens and resistant strains. Therefore, this study examined the antibiotic resistance profiles of bacteria isolated from loggerhead sea turtles hospitalised in a rescue centre of Southern Italy over a 9-year period. Resistance to ceftazidime, doxycycline, enrofloxacin, flumequine, gentamicin, oxytetracycline and sulfamethoxazole-trimethoprim was evaluated for 138 strains isolated from the clinical samples or organs of 60 animals. Gram-negative families were the most isolated: *Vibrionaceae* were predominant, followed by *Shewanellaceae*, *Pseudomonadaceae*, *Enterobacteriaceae* and *Morganellaceae*. These last three families exhibited the highest proportion of resistance and multidrug-resistant strains. Among the three Gram-positive families isolated, *Enterococcaceae* were the most represented and resistant. The opportunistic behaviour of all the isolated species is particularly concerning for diseased sea turtles, especially considering their resistance to commonly utilised antibiotics. Actually, the multiple antibiotic resistance was higher when the sea turtles were previously treated. Taken together, these findings highlight the need to improve antimicrobial stewardship and monitor antibiotic resistance in wildlife, to preserve the health of endangered species, along with public and environmental health.

## 1. Introduction

Antimicrobial resistance (AMR) is one of the major problems challenging human and animal health in our century. The discovery and development of antibiotics was among the most important and successful cornerstones in the healthcare field. For many years, AMR has been contrasted by the introduction of new molecules on the market. However, in recent years, the development of new antibiotics has slowed down, and this, along with other aspects, has allowed an increase in antibiotic resistance [1,2]. Actually, antibiotic resistance is an ancient phenomenon, because it is the result of the adaptation of organisms to their environment; on the one hand, bacteria develop resistance mechanisms to defend themselves and survive; on the other, many antibiotics are naturally occurring molecules, and for this reason, intrinsic resistance may exist [3,4]. However, the current worldwide emergency mainly refers to acquired resistance, which is largely caused by the improper or massive use of antibiotics and which threatens global health [5,6].

Indeed, the appearance and dissemination of AMR has direct and indirect repercussions on different sectors involving humans and animals. In clinical settings, AMR compromises the ability to effectively treat infections, forces the use of last-resort molecules, increases the chances of complications in vulnerable populations, and prolongs treatment or hospitalisation periods, ultimately resulting in inflated healthcare costs [7,8]. In livestock farming, all of this translates into serious economic losses, since reductions in feed conversion, growth rates and reproductive performances contribute to decreased productivity, especially in low–middle income countries, thus widening the gap with other countries [5,9].

For wildlife conservation, the consequences of AMR have probably been overlooked and generally considered minor, compared to the particular interest raised by the potential role of wild animals as reservoirs of antibiotic-resistant bacteria (ARB), especially in relation to possible risks for public health [10,11]. Although wildlife is a recognised part of resistance dynamics, as suggested by several reports of ARB in different species and habitats [12], the origins, routes and implications of this dynamics are still not fully understood [10,13]. Wild animals are unlikely to be subjected to antibiotic treatment; therefore, research has been oriented towards their acquisition of ARB from the environment. In this regard, numerous studies have suggested that increasing interactions between humans, domestic animals and wild animals enhance the risk of transferring ARB to wildlife, and vice versa [11,12,14]. Specifically, the increase in human pressure (including the loss and fragmentation of habitats, the release of agricultural and urban wastewater, climate change, etc.) facilitates the dissemination of pathogens and antibiotic resistance in the environment. Aquatic ecosystems are considered particularly impacted, also, considering the role played by water in the dispersion of antibiotics and ARB [13,15,16]. Surface water, wastewater and recreational water have been previously monitored for the presence of ARB, and several environments (e.g., fish farms, animal manure, hospitals, spill events) have been identified as potential sources of antibiotic residues and ARB. Through seepage or sewage, resistance elements can reach and contaminate underground waters, lagoons or effluents, ending up in marine habitats and resulting in the appearance and transfer of resistance in marine organisms [15,16,17].

From a different point of view, some wild species, due to their characteristics and responses to environmental changes, could serve as sentinels of challenged habitats or anthropogenic stressors, and potentially provide an early warning system for ecosystem and public health [18]. In the marine environment, seabirds, marine mammals and sea turtles have long been used as sentinels of marine pollution due to their lifespan, global distribution, trophic level or habitat use [18,19,20,21]. Specifically, loggerhead sea turtles are long-living animals distributed in warm and temperate seas throughout the world, and they feed at progressively higher trophic levels as they grow, with the consequent tendency of bio-accumulation [22,23]. Moreover, these species frequently inhabit near-shore ecosystems, which are the most impacted by anthropogenic activities, and are susceptible to similar noxious agents as other animals and humans (pathogens, toxins and chemical compounds). Actually, the exposure to polluted areas might be increased by the complex life cycle of sea turtles, because they utilise different habitats as nesting sites, migratory routes or foraging grounds, thus multiplying the risk of contact with and acquisition of ARB, possibly through food, seawater or even sand [23,24,25]. In the Mediterranean Sea, antibiotic resistance has been recently detected in several bacteria isolated from wild and captive sea turtles, both healthy and diseased [15,26,27].

The majority of bacteria in sea turtles are considered opportunistic pathogens; therefore, the presence of ARB is especially concerning for diseased or captive animals, which might be at greater risk of secondary infections [28]. In these cases, antibiotic resistance might limit therapeutic options, interfere with the rehabilitation process, and delay the reintroduction to nature, ultimately affecting the health of individuals and the population [10,29]. Within this context, rescue and rehabilitation centres play a significant role for sea turtle conservation, by preserving the health of juvenile and adult turtles, which are the most commonly rescued and the most important for population stability, due to their reproductive value [30,31,32,33].

Loggerhead sea turtles are distributed throughout the Mediterranean basin, with an estimated population of 1,201,845 (95% CI 838,864–1,548,280) [34]. The distribution of *C. caretta* is widespread along all the coasts of the Italian peninsula. However, the Adriatic and Ionian areas are important for the developmental stages of juveniles [23]. Instead, the Tyrrhenian coast, in particular the Campania region, provides foraging and nesting grounds [22,35].

In the Campania region, in Southern Italy, a sea turtle stranding, conservation and monitoring network has been in place for the last decades, involving several partners. Among them, the Istituto Zooprofilattico Sperimentale del Mezzogiorno (IZSM) and the Stazione Zoologica Anton Dohrn (SZN) are responsible for healthcare and research activities on dead and live sea turtles, respectively. Therefore, this study aimed at investigating the antibiotic resistance profiles of bacteria isolated from diseased and captive sea turtles, through a retrospective analysis conducted on loggerhead sea turtles (*Caretta caretta*) rescued and hospitalised at the rescue centre of SZN “Turtle Point” (TP, Portici, Italy).

## 2. Materials and Methods

### 2.1. Study Population

This study included bacterial strains belonging to 60 Mediterranean loggerhead sea turtles (Table S1), admitted at the rescue centre of SZN (TP) over a period of 9 years (2015–2023). All animals were alive at the time of recovery and were found adrift (*n* = 35), stranded (*n* = 18) or bycaught (*n* = 7) off the Italian coasts. Sea turtles recovered from Campania waters (*n* = 31) were immediately transported to the TP, while others (*n* = 29) were transferred to the TP from other Italian rescue centres (e.g., Lazio, Sicily) at a later stage, in order to access long-term or specialised care.

Upon admission, the following morphometric parameters were measured: body weight (range = 0.05–68.95 kg; mean ± SD = 22.82 ± 18.45 kg); curved carapace length (range = 7.8–84 cm; mean ± SD = 54.8 ± 17.7 cm); curved carapace width (range = 7.5–86.5 cm; mean ± SD = 50.6 ± 16.9 cm); straight-line carapace length (range = 6.6–76 cm; mean ± SD = 47.9 ± 17.7 cm); and straight-line carapace width (range = 5.8–61.7 cm; mean ± SD = 39.5 ± 14.1 cm).

The responsible veterinarian examined each animal to evaluate the cause of hospitalisation, the type and severity of lesions, and treatment and rehabilitation protocols. Samples for bacteriological analyses and antibiotic susceptibility testing (AST) were collected depending on the lesions and veterinary diagnoses, as described below, in order to rule out or prevent infections. However, the sampling did not always occur upon admission, since lesions ascribable to potential infections could have manifested throughout the hospitalisation period. Additionally, in a few cases, samples were collected only with the purpose of medical clearance just before reintroduction into nature.

Turtles that died upon admission at the TP or during hospitalisation, before sampling (*n* = 10), were sent to the IZSM to conduct post-mortem examinations and investigate the causes of death. In these cases, bacteriological analyses and AST were carried out on the organs collected during the necropsy, as detailed below.

Since the timing of sample collection differed between animals, the examined individuals were categorised as treated and untreated sea turtles: the former group consisted of animals submitted to antibiotic treatment before sample collection (*n* = 20); the latter consisted of animals not submitted to antibiotic treatment before sample collection, including those that did not receive any antibiotic treatment at all (*n* = 40).

### 2.2. Sample Collection

From living sea turtles, clinical samples were collected at the TP, according to the type and site of lesions and to the suspected diagnoses, using sterile cotton-tipped swabs or needle aspiration.

Specifically, in cases of external lesions (e.g., carapace fractures, flipper wounds, fistulas, skin discolorations) swabs samples were collected from the inner part of the lesion, following superficial curettage. In cases of ocular lesions, swab samples were obtained from the conjunctival fornix after gently distancing the eyelids. Whenever possible, a small amount of necrotic tissue, if present within the affected site, was also collected from the inner part of the lesions. In suspected cases of seawater aspiration, swab samples were collected from the glottis, after opening the rhamphotheca with a previously disinfected PVC tube. When coelomitis or gastrointestinal perforations were suspected, coelomic fluid was collected by needle aspiration through the prefemoral fossa, after skin disinfection with 4% chlorexidine digluconate. In other suspected cases of internal lesions (e.g., abscesses), needle aspiration was performed to collect a small amount of tissue from the inner part of the lesion, after disinfecting the surrounding skin with 4% chlorexidine digluconate. In only one case, lymphatic fluid was erroneously collected during blood sampling and sent for bacteriological analyses. In cases of the absence of lesions, uncertain diagnoses or pre-release medical clearance, oropharyngeal and cloacal sampling were performed as a screening method. For oropharyngeal samples, a swab was gently brushed over the oral mucosa, after opening the rhamphotheca (with a previously disinfected PVC tube). For cloacal samples, a swab was gently inserted and rotated into the cloaca (1–5 cm, depending on the animal size), after rinsing the pericloacal area with 4% chlorexidine digluconate.

All procedures involving living animals were included in the clinical examination and diagnostic investigations of the rescued sea turtles, in accordance with the authorisation by the Ministry of the Environment (Protocols n. 0042848/PNM 9 August 2013, n. 0024471/PNM 22 November 2016, n. 0000992/PNM 22 January 2020, n. 0020551/PNM 13 February 2023).

From dead sea turtles, organs were sampled at the IZSM during post-mortem examination, performed according to Wyneken, 2001 [36] and Poppi and Marchiori, 2013 [37]. Briefly, the external examination included species identification, the collection of morphometric data, the classification of condition and nutritional code, and the evaluation of possible external lesions. The internal examination allowed access to and inspection of the coelomic organs, by removing the plastron and the shoulder blades, as well as the brain and salt glands, by separating and opening the skull. During the necropsy, from all sea turtles, the liver, kidneys, spleen, intestine, brain, and lungs were routinely sampled independently from any associated lesions, whereas other organs were sampled only upon the presence of lesions. The surface of each target organ was scalded with a spatula and cut with a sterile blade, in order to collect a swab sample from the inner part, avoiding contamination

All collected samples were transferred and processed at the IZSM for bacterial isolation, identification and antibiotic susceptibility testing.

### 2.3. Bacterial Isolation and Identification

All samples were streaked on Blood Agar (BA) (Biolife, Monza, Italy) and Tryptone Soy Agar with 2% NaCl (TSAs) (Microbiol srl, Cagliari, Italy), and the plates were incubated at 25 °C ± 1 °C for 24–72 ± 3 h under aerobic conditions; contrarily, the plates from the intestine were incubated only under anaerobic conditions. Bacterial colonies grown on BAs and TSAs were selected according to their morphology, isolated and tested with oxidase (Oxoid, Thebarton, South Australia), catalase (bioMérieux, Marcy-l’Etoile, France), motility tests and Gram stain [38,39]. The strains were subsequently confirmed to the genus or species level through miniaturised biochemical identification systems, such as API^®^ and VITEK2^®^ (bioMérieux, Marcy-l’Etoile, France).

### 2.4. Antibiotic Susceptibility Testing

All identified strains were subjected to AST using the disk diffusion method (Kirby–Bauer), in accordance with the criteria established by the Clinical & Laboratory Standards Institute (CLSI) documents [40,41]. Specifically, an inoculum of 0.5 McFarland was prepared for each bacterial strain. Within 15 min of preparing the inoculum, a swab was dipped into the suspension and streaked over the entire surface of the plate. Different culture media were chosen, according to the strain growth requirements: Mueller Hinton Agar (MH) (Microbiol srl, Cagliari, Italy) was used for *Enterobacteriaceae*, *Pseudomonadaceae* and other Gram-negative bacteria; Mueller Hinton + 5% Blood (MHB) was used for Gram-positive bacteria; and Mueller Hinton + 1.5% NaCL (MHS) for halophilic bacteria such as *Vibrionaceae* [40,42,43].

Given the clinical application, the antibiotics to be tested were chosen among the molecules which are commonly used for sea turtles, selecting the following antibiotic disks (Oxoid, Basingstoke, UK): ceftazidime (CAZ, 30 µg); doxycycline (DO, 30 µg); enrofloxacin (ENR, 5 µg); flumequine (UB, 30 µg); gentamicin (CN, 10 µg); oxytetracycline (OXY, 30 µg); and sulfamethoxazole-trimethoprim (SXT, 25 µg). Each antibiotic disk was picked up with sterile forceps and placed on the plate. All plates were incubated at 22 °C ± 1 °C for 24 ± 3 h. The diameter of the inhibition zone was measured with a calliper, and the results were classified as susceptible (S), intermediate (I) and resistant (R), following clinical breakpoints for aquatic animals as suggested by the CLSI documents [40,41].

For each strain, the multiple antibiotic resistance (MAR) index was calculated as a ratio between the number of resistances and the number of tested antibiotics [44]. Additionally, strains exhibiting resistance to at least three different classes of antibiotics were defined as multidrug-resistant (MDR), in accordance with Magiorakos et al. (2012) [45].

### 2.5. Data Analysis

All data were entered and analysed in an Excel spreadsheet (Microsoft, Redmond WA, USA). The proportion of MDR strains and the MAR indices were compared between treated and untreated sea turtles. Statistical analyses were performed with chi-square and Fisher exact tests, setting significance level at *p* < 0.05 and using MedCalc^®^ (version 10.3.2.0, Mariakerke, Belgium).

## 3. Results

### 3.1. Bacterial Isolation and Identification

**Figure 1:**
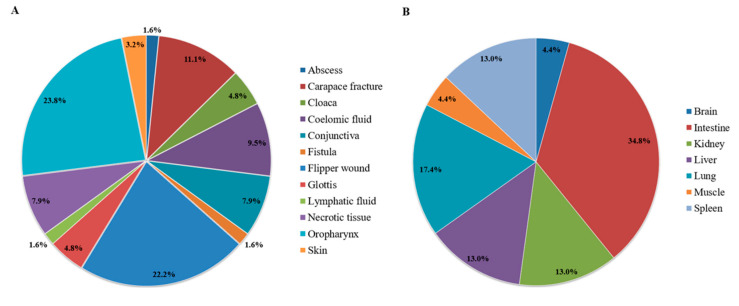
Sampling sites resulting in bacterial isolation, expressed as percentages over the total of 63 clinical samples (**A**) and 23 organs (**B**), collected from 60 loggerhead sea turtles.

This study included a total of 138 bacterial strains isolated from 60 sea turtles, 50 of which were live turtles and 10 of which were dead. The vast majority (123/138; 89.1%) of bacteria were isolated from clinical samples (*n* = 63), whereas the remaining ones (15/138; 10.9%) were isolated from organs (*n* = 23). The different sampling sites that resulted positive to bacterial isolation are summarised in Figure 1, distinguished between clinical samples (Figure 1A) and organs (Figure 1B). It is important to mention that a bacterial strain could have been cultured from different samples of the same turtle, and, vice versa, multiple bacterial strains could have been cultured from the same sample.

**Figure 2:**
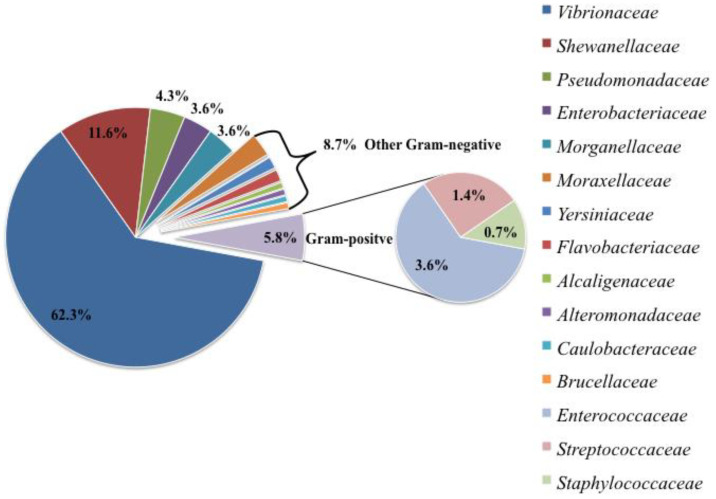
Bacterial families isolated from clinical samples and organs of 60 loggerhead sea turtles.

Gram-negative organisms represented almost the entirety of the isolated bacteria (130/138; 94.2%), whereas Gram-positive ones accounted for a small portion of the total amount of strains (8/138; 5.8%). In particular, 15 bacterial families were cultured and identified, 12 of which belonged to Gram-negative and 3 to Gram-positive organisms. Within Gram-negative organisms, the most represented families were Vibrionaceae (86/138; 62.3%) and *Shewanellaceae* (16/138; 11.6%), followed by Pseudomonadaceae (6/138; 4.3%), Enterobacteriaceae (5/138; 3.6%) and *Morganellaceae* (5/138; 3.6%). The other Gram-negative families (i.e., *Alcaligenaceae*, *Alteromonadaceae*, *Brucellaceae*, *Caulobacteraceae*, *Flavobacteriaceae*, *Moraxellaceae*, *Yersiniaceae*) were less represented (each <3%) and together constituted only 8.7% of the total amount of strains (12/138), as summarised in Figure 2.

Within Gram-positive organisms, Enterococcaceae (5/138; 3.6%), Streptococcaceae (2/138; 1.4%) and Staphylococcaceae (1/138; 0.7%) were the only represented families (Figure 2).

Bacterial families were isolated with different frequencies from clinical samples and organs. In particular, *Vibrionaceae*, *Pseudomonadaceae* and *Enterococcaceae* were more frequently isolated from clinical samples (94.2%, 83.3 and 80%, respectively) than organs (5.8%, 16.7% and 20%, respectively). Contrarily, *Enterobacteriaceae* and *Morganellaceae* were more frequently isolated from organs (80%) than clinical samples (20%). The remaining bacterial families, including *Shewanellaceae*, were exclusively isolated from clinical samples.

**Figure 3:**
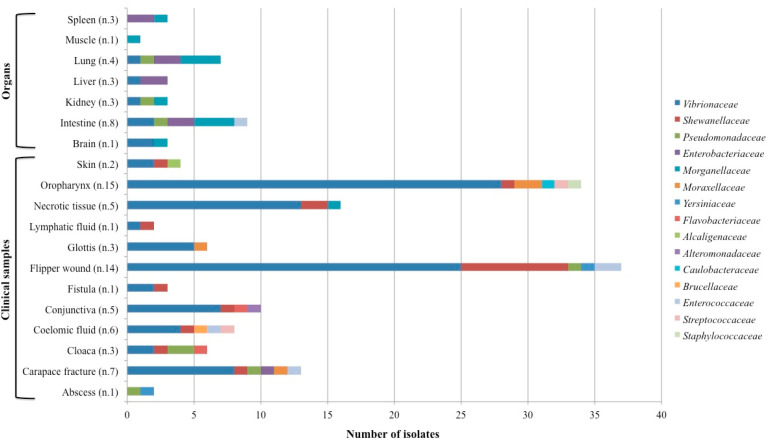
Number of strains in clinical samples and organs collected from 60 loggerhead sea turtles. Reported in brackets is the number of samples examined for each site.

Focusing on the sampling sites (Figure 3), swabs from the oropharynx, flipper wounds, necrotic tissue and carapace fractures resulted in the isolation of numerous strains, predominantly from the *Vibrionaceae* family. Similarly, *Shewanellaceae* and *Enterococcaceae* were mainly isolated from flipper wound swabs, whereas *Pseudomonadaceae* were slightly more frequent from cloacal swabs. Contrarily, *Enterobacteriaceae* and *Morganellaceae* were mainly isolated from the intestine and lung, followed by the liver and spleen.

Regarding bacterial identification (Table S2), 21 species of the *Vibrionaceae* family were confirmed: *Vibrio alginolyticus* (*n* = 26), *Vibrio harveyi* (*n* = 22), *Vibrio fluvialis* (*n* = 5), *Vibrio navarrensis* (*n* = 5), *Vibrio diazotrophicus* (*n* = 3), *Vibrio anguillarum* (*n* = 1), *Vibrio furnissii* (*n* = 1), *Vibrio gallicus* (*n* = 1), *Vibrio mediterranei* (*n* = 1), *Vibrio orientalis* (*n* = 1), *Vibrio parahaemolyticus* (*n* = 1), *Vibrio pelagius* (*n* = 1), *Vibrio pelagius* II (*n* = 1), *Vibrio scophthalmi* (*n* = 1), *Vibrio splendidus* I (*n* = 1), *Vibrio splendidus* II (*n* = 1), *Vibrio tubiashii* (*n* = 1), *Vibrio xuii* (*n* = 1), *Photobacterium damselae* subsp. *damselae* (*n* = 4), *Photobacterium swingsii* (*n* = 1) and *Grimontia hollisae* (*n* = 1). In six additional cases, the strains were not successfully identified to the species level; therefore, they were reported as *Vibrio* spp. (*n* = 4), *Photobacterium* spp. (*n* = 1) and *Aliivibrio* spp. (*n* = 1). Within *Shewanellaceae*, the only two isolated species were *Shewanella algae* (*n* = 13) and *Shewanella putrefaciens* (*n* = 3). Among *Pseudomonadaceae*, the isolated species were *Pseudomonas aeruginosa* (*n* = 2), *Pseudomonas putida* (*n* = 2), *Pseudomonas fluorescens* (*n* = 1) and *Pseudomonas mendocina* (*n* = 1). The species belonging to the *Enterobacteriaceae* family were *Citrobacter freundii* (*n* = 4) and *Enterobacter cancerogenus* (*n* = 1), whereas the only species within *Morganellaceae* was *Morganella morganii* subsp. *morganii* (*n* = 5).

Among Gram-positive families, *Enterococcus faecalis* (*n* = 3), *Enterococcus gallinarum* (*n* = 1) and *Vagococcus fluvialis* (*n* = 1) were the isolated species within *Enterococcaceae*, whereas *Lactococcus garviae* (*n* = 2) and *Staphylococcus sciuri* (*n* = 1) were the only species belonging respectively to *Streptococcaceae* and *Staphylococcaceae*.

### 3.2. Antibiotic Susceptibility Testing

**Figure 4:**
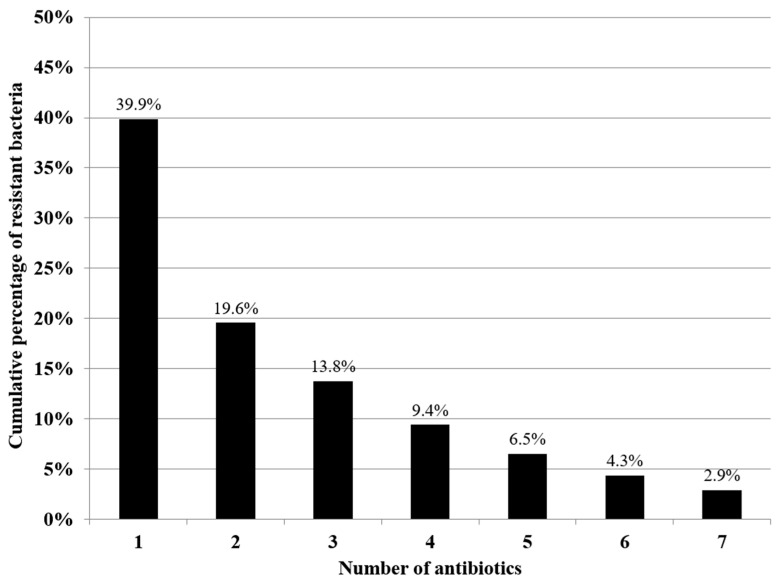
Cumulative percentages of resistance to one or more antibiotics of 138 bacterial strains isolated from clinical samples and organs of 60 loggerhead sea turtles.

The antibiotic resistance profile of all the bacterial strains was evaluated. Less than half of the bacterial strains (55/138; 39.9%) exhibited resistance to one or more of the tested antibiotics, whereas only a small number of strains (4/138; 2.9%) exhibited simultaneous resistance to all of the tested antibiotics (Figure 4).

Bacterial strains exhibited different profiles of antibiotic resistance, which are detailed in Table 1; in particular, the highest rates of resistance were detected towards ceftazidime (17.5% of the strains) and gentamicin (16.8%). For the other tested antibiotics, similar percentages of resistant strains were found for doxycycline and sulfamethoxazole-trimethoprim (respectively, 13.8% and 13.1%) and for enrofloxacin and oxytetracycline (both 12.3%), whereas the lowest percentage was found for flumequine (11.1%). Relatively high percentages of strains (always above 64%) were susceptible to the tested antibiotics, with the highest value (85.4%) detected for sulfamethoxazole-trimethoprim (Table 1).

When considering the main bacterial families (contributing at least 3% of the total amount of strains), *Vibrionaceae* and *Shewanellaceae* were those with the lowest percentages of resistant strains to the selected antibiotics, compared to the other families. Specifically, 10.5% of *Vibrionaceae* strains were resistant to gentamicin and even lower percentages were resistant to other antibiotics: sulfamethoxazole-trimethoprim (8.2%), ceftazidime (8.1%), doxycycline (7%), oxytetracycline (7%), enrofloxacin (5.8%) and flumequine (1.2%). Similarly, 18.8% of *Shewanellaceae* strains were resistant to doxycycline, while the resistance rates for the other tested antibiotics were between 6.3 and 6.7%. *Pseudomonadaceae* was the family with the highest rate of resistance to sulfamethoxazole-trimethoprim (66.7% of strains), whereas lower rates of resistance were detected for ceftazidime, doxycycline, enrofloxacin, oxytetracycline (50%), flumequine and gentamicin (33.3%). As regards *Enterobacteriaceae*, the resistance rates ranged between 40% (i.e., flumequine, oxytetracycline, sulfamethoxazole-trimethoprim) and 60% (i.e., ceftazidime, doxycycline, enrofloxacin, gentamicin). *Morganellaceae* was the family with the highest rates of resistance (80% of strains) towards four of the seven selected antibiotics (i.e., doxycycline, enrofloxacin, flumequine and oxytetracycline); relatively lower resistance rates were detected towards sulfamethoxazole-trimethoprim and gentamicin (60.0%), whereas only 20% of strains were resistant to ceftazidime.

Among the Gram-positive families, no strain was resistant to doxycycline or oxytetracycline, and no strain within *Enterococcaceae* was resistant to sulfamethoxazole-trimethoprim either. On the contrary, all *Enterococcaceae* were resistant to gentamicin and 75% of strains were resistant to ceftazidime, whereas the resistance rates towards enrofloxacin and flumequine were 20% and 60%, respectively (Table 2).

Concerning simultaneous resistance to multiple antibiotics, the MAR indices ranged from 0 to 1, with a MAR index for all bacterial strains of 0.14, which varied between bacterial families. Specifically, the highest MAR index was attributed to the family *Morganellaceae* (0.66), followed by *Enterobacteriaceae* (0.51), *Pseudomonadaceae* (0.48) and *Enterococcaceae* (0.31). On the contrary, *Vibrionaceae* and *Shewanellaceae* were the families with the lowest MAR indices (respectively, 0.07 and 0.08).

Additionally, eleven strains were defined as MDR: three belonging to *Enterobacteriaceae* (*C. freundii*); three to *Morganellaceae* (*M. morganii* subsp. *morganii*), two to *Pseudomonadaceae* (*P. aeruginosa* and *P. putida*); two to *Vibrionaceae* (*V. alginolyticus*); and one to *Shewanellacae* (*S. algae*).

The MAR indices and the proportions of MDR strains were compared between the two groups of sea turtles classified as treated and untreated. The MAR index in the treated group was significantly higher than the MAR index in the untreated group (respectively, 0.29 and 0.07; χ^2^ = 77.917; DF = 1; *p* < 0.01). Similarly, the proportion of MDR strains was significantly higher in the treated group compared to the untreated group (respectively, 20.9% and 2.1%; χ^2^ = 14.3; DF = 1; *p* < 0.01).

## 4. Discussion

Loggerhead sea turtle bacteria have attracted increasing attention, to learn more about their role as part of normal microbiota or as pathogens [27,46,47]. In the current study, the bacterial strains isolated from rescued loggerhead sea turtles were considered opportunistic pathogens, in accordance with Ebani [28]. Indeed, the health status of the examined turtles was compromised for different reasons, including the underlying conditions leading to hospitalisation and the stress related to captivity.

Generally, bacterial entry has been hypothesised from the environment through dermal, oral or cloacal routes [48]. In accordance with this hypothesis, the strains described in the present results were mainly isolated from sites exposed to the external environment, such as open injuries (such as limb wounds or carapace fractures) and mucosae (such as the oropharyngeal cavity and the conjunctiva).

The greater presence of Gram-negative bacteria compared to Gram-positive ones is in agreement with previous studies [25,27,49]. However, the predominance of *Vibrionaceae* over other bacterial families has not always been reported [48,50], as *Enterobacteriaceae* have usually been the most commonly isolated [26,51].

*V. alginolyticus* and *V. harveyi* were the most frequently isolated Vibrionaceae species, in line with previous studies [48,52]. Both species are normally present in aquatic environments, but have also been associated with lesions in sea turtles, such as dermatitis, enteritis and pneumonia [28,53]. Moreover, these species are considered important pathogens of marine animals and potential zoonotic agents [54,55]. Within Vibrionaceae, it is worth mentioning the isolation of *P. damselae damselae* and *V. parahaemolyticus*, despite the limited number of strains, because of their recognised pathogenicity in humans and other animals [49,56,57].

The low rates of antibiotic resistance detected among the isolated *Vibrionaceae* are in line with some previous studies regarding aquaculture and coastal waters [58,59] but in contrast with others documenting higher resistance rates [60,61]. These variations have been usually related to the strain origins, the nutrient levels in the waters, or the use of antibiotics in different countries. For instance, sulphonamides have been widely used in Asia [59], whereas oxytetracyclines have been the most commonly licensed for European aquaculture, followed by flumequine [58]. Additionally, tetracyclines and fluoroquinolones are the antibiotics of choice to treat severe vibriosis in human and veterinary practice [58,60]. In sea turtles, *V. alginolyticus* has been described as the *Vibrio* species with the highest rates of antibiotic resistance [52], as also highlighted by the two MDR strains isolated in the present study.

*Shewanellaceae* have been frequently reported among the predominant bacterial families in sea turtles, usually as part of the normal microbiota [26,62]. However, the isolation of S. algae, and to a lesser extent of *S. putrefaciens*, mainly from flipper wounds and necrotic tissue, might underline an opportunistic role in the development of secondary infections [63,64]. Despite the low antibiotic resistance rates of *Shewanellaceae*, almost 20% of strains were resistant to doxycycline, and one MDR strain was found resistant to five out of seven antibiotics (except for gentamicin and sulfamethoxazole-trimethoprim). Indeed, the genus *Shewanella* has been increasingly reported as responsible for several infections and the appearance of antibiotic resistance [65]. In particular, immunocompromised individuals are at risk of developing soft tissue infections, and treatment might be limited by the presence of resistance genes to β-lactams and quinolones, which are commonly utilised in human medicine [65].

*Pseudomonadaceae* have been commonly isolated from sea turtles, in particular from cloacal swabs [25,51,66], similarly to our results. However, *Pseudomonas* has been found in association with different lesions, including respiratory and ocular infections [28,53]. The antibiotic resistance of the *Pseudomonadaceae* family, in particular of *P. aeruginosa*, has always received particular attention and has been investigated in different matrices [67,68,69]. In the present study, Pseudomonadaceae were the third family in terms of rates of resistance and MDR strains, similarly to previous results that reported *Pseudomonas* species as the ones exhibiting resistance to the highest numbers of antibiotics, both in green and loggerhead sea turtles [70,71]. These resistance properties could also be transferred through mobile elements to other bacterial species, which is particularly concerning, considered the ubiquitous nature of *Pseudomonas* species and their potential as opportunistic pathogens for humans and animals alike [67].

Although *Enterobacteriaceae* were not the dominant family within the Gram-negative bacteria here isolated, contrarily to previous reports [26,51], *Citrobacter* is confirmed as one of the most common genera described within this family [72,73]. Indeed, *Citrobacter* species are well documented sea turtle pathogens, responsible for several lesions [53,74,75], especially in weak and captive turtles [26,27]. Additionally, high levels of antibiotic resistance have frequently been detected in Citrobacter, and Enterobacteriaceae in general, consistent with the present results [71,72,73]. These findings support the existing literature regarding the role of Enterobacteriaceae in the circulation of antibiotic resistance among environmental, commensal and pathogenic bacteria [72,76]. Enterobacteriaceae have an extraordinary ability to acquire resistance from the environment, especially when exposed to antibiotics (in particular, β-lactams) [77]. Moreover, this bacterial family is considered the main carrier of extended-spectrum β-lactamase (ESBL)-encoding genes, which could be transferred with other resistance genes through large mobile elements, thus promoting the occurrence of MDR [78]. Given the abundance of ESBL-producing Enterobacteriaceae and resistance elements described in aquatic environments (e.g., wastewater, freshwater, estuaries and coastal waters), the exchange of resistance from Enterobacteriaceae to other bacteria, and vice versa, could be enhanced in contaminated waters [17,79]. When this gene flow involves pathogenic species, *Enterobacteriaceae* might serve as mediators between aquatic and clinical settings, with the consequent risk of treatment failure [76,80].

*Morganellaceae* are a relatively new family, composed of species formerly included within the Enterobacteriaceae [81]; therefore, some reports still refer to *M. morganii* as a member of Enterobacteriaceae. In addition to isolation from oropharyngeal and cloacal samples [63,73] M. morganii has been detected in leatherback sea turtles with intestinal diverticulitis [82], and it has been associated with wounded and rehabilitated sea turtles, like those examined in our study [27,72]. Consistent with previous reports from sea turtles in areas subjected to elevated anthropic pressure, such as the Mediterranean Sea and the Great Barrier Reef [51,72], Morganellaceae isolated in this work exhibited the greatest rates of resistance towards the selected antibiotics, with the exception of ceftazidime. Contrarily, Morganellaceae isolated from sea turtles of the North-East Atlantic only exhibited intermediate resistance to carbapenems, probably because of the more pristine environment characterizing the island of Maio [63]. A high proportion of MDR strains was observed within this family, although this finding has been mainly ascribed to Morganellaceae’s intrinsic properties [45,72].

Regarding Gram-positive microorganisms, far fewer reports are currently available in sea turtles, compared to Gram-negative; nevertheless, the strains found in this study belonged to the *Enterococcaceae*, *Streptococcaceae* and *Staphylococcaceae* families, as previously described [28,62]. Similarly to Gram-negative bacteria, studies carried out on Gram-positive bacteria usually refer to many species as part of the normal microbiota of sea turtles [66,83,84]. However, these species have also been reported as potential pathogens, alone or in association with Gram-negative bacteria [53,75]. Among Gram-positive families, *Enterococcaceae* was the most frequently isolated within this study; in particular, *Enterococcus faecalis* is confirmed as one of the most common enterococci in sea turtles [28,85]. Although usually present in the marine environment, *E. faecalis* has been isolated from sea turtles with respiratory and skin infections, osteomyelitis and lesions of the bladder, liver, lungs and muscles [49,86,87]. Antibiotic resistance has often been observed in *Enterococcaceae*; in particular, the strains isolated in this study were found resistant to cephalosporins, aminoglycosides and quinolones, in accordance with previous reports [86,87,88]. Contrarily, the *Enterococcus* strains here isolated were not resistant to tetracyclines, as described in different marine species [85,87,89]. The medical management of sea turtles infected by Enterococcus might prove challenging, as it often requires a long-term or combined use of multiple antibiotic classes. In particular, the wide use of cephalosporins in sea turtles might contribute to the emergence of resistant enterococci [86,87]. E. faecalis is also considered responsible for many human enterococcal infections, potentially resulting in lethal systemic disease when improperly treated [90]. Within the other two Gram-positive families detected in our study, *Staphylococcus sciuri* and *Lactococcus garviae* (*Staphylococcaceae* and *Steptococcaceae*, respectively) have seldom been reported in sea turtles. Specifically, *S. sciuri* has been detected in blood samples of a green turtle affected by fibropapillomatosis [48], whereas *L. garviae* has been isolated from the liver and kidney of a loggerhead sea turtle and from the cloacal sample of a green turtle [49,89]. Antibiotic resistance was detected toward ceftazidime (*S. sciuri* and 1/2 *L. garviae*), flumequine (2/2 *L. garviae*) and sulfamethoxazole-trimethoprim (1/2 *L. garviae*), similarly to previous reports [89,91].

The finding of significantly higher MAR indices and a proportion of MDR strains among the bacteria isolated from sea turtles that received antibiotic treatment, compared to those that did not receive antibiotics (or that initiated treatment following sample collection), might suggest that antibiotic treatment had an influence on antibiotic resistance. Indeed, antibiotic treatment could promote resistance through mutations, resistant genes or strain replacement [92]. In particular, reduced dosages, limited tissue permeability and incomplete treatment schedules are known factors contributing to the appearance of antibiotic resistance [93,94]. However, even when antibiotic therapy is accurately prescribed and performed, it could induce the emergence of resistance, predominantly by the selection of resistant strains within the microbial communities of the host, instead of de novo evolution [92]. This is especially true for wound pathogens, such as the ones that were mainly isolated in the present study. On the other hand, some antibiotics, especially quinolones and β-lactams, are involved in the emergence of antibiotic resistance through different mechanisms of mutagenesis, leading to mutations that might be maintained in the host microbiota or be transferred to pathogenic agents [94,95,96]. Additionally, quinolones and cephalosporins are among the most commonly used antibiotics in sea turtles; however these classes include molecules of the Watch group in the AWaRe classification, which are first- and second-choice antibiotics indicated for specific infections and more susceptible to be the target of antibiotic resistance [97].

Therefore, the presence of ARB in sea turtles represents a risk to their health, as it could restrict antibiotic options to treat bacterial infections and potentially compromise the rehabilitation success [27,98]. From a wider perspective, this risk might be extended to humans and other animals, since sea turtles are a potential carrier of zoonotic agents [99,100,101,102]. Zoonoses have been reported to spill over from terrestrial to marine species and back, outlining marine animals not only as victims but also as vectors [52,91,103,104]. Ecotourism and conservational efforts might contribute to the expansion of sea turtle zoonoses, although the major concerns derive from interactions with captive or dead sea turtles and the consumption of turtle products (still practiced in some areas) [52,104,105]. Therefore, some populations (marine animal researchers, rehabilitators, trainers, veterinarians and volunteers) might be at greater risk of transmission through occupational exposure [91,103]. The staff working at the TP was not tested for the presence of ARB; however, biosecurity measures are commonly adopted in the centre, and to our knowledge there were no reports of bacterial infections through occupational exposure during the period of study. The dissemination of antibiotic resistance in pathogenic bacteria poses additional risks for public health, and it becomes particularly alarming when it involves antibiotics commonly used in human medicine or last-resort molecules for the treatment of serious infections, as it reduces their effectiveness and thus any chance of a favourable outcome [10,29,63].

## 5. Conclusions

Bacterial infections affecting sea turtles are mainly caused by opportunistic agents, usually present in the surrounding environment or as commensal microorganisms. The results of this study provide valuable information on antibiotic resistance in bacteria affecting hospitalised sea turtles. Even if this study does not assess possible variations in antibiotic resistance over time, the present findings cover almost a decade and could represent an appropriate baseline for future investigations. Moreover, these data could be useful to organisations and healthcare and research bodies involved in the rehabilitation and management of sea turtles and potentially other aquatic species.

It is of fundamental importance to improve antimicrobial stewardship in wildlife facilities: susceptibility testing should be performed before initiating antibiotic therapy; narrow-spectrum antibiotics should be preferred to broad-spectrum antibiotics; Access group antibiotics should be prioritised over other groups; and a treatment schedule should be strictly followed [106].

Additional measures to prevent and treat infections in sea turtles and other animals include ensuring the adequate hygienic conditions of the surrounding environment, such as the rearing water [107], and investigating alternative options to antibiotics, such as phage therapy in sea turtles [108,109].

Given the presence of potentially resistant zoonotic bacteria, the adoption of these recommendations and the continuous monitoring of antibiotic resistance will be beneficial in preserving these endangered species, as well as public health and the environment, within a wider One Health perspective. Further research and a close collaboration among different professional figures are strongly needed to clarify the possibilities and mechanisms of antibiotic resistance dissemination across environments, animals and humans. Moreover, molecular methods, which were not applied in the present study, could contribute to the determination of resistant genes, the identification of their sources and the elucidation of their transmission dynamics, in order to infer the potential effects on all the sectors involved.

## Figures and Tables

**Table 1:** Antibiotic resistance of 138 bacterial strains isolated from clinical samples and organs of 60 loggerhead sea turtles.

Antibiotics	Phenotypic Profile of Bacterial Strains (%)		
	Susceptible	Intermediate	Resistant
CAZ	78.1%	4.4%	17.5%
DO	76.8%	9.4%	13.8%
ENR	74.6%	13.0%	12.3%
UB	75.6%	13.3%	11.1%
CN	66.4%	16.8%	16.8%
OXY	64.5%	23.2%	12.3%
SXT	85.4%	1.5%	13.1%

CAZ = ceftazidime (30 µg); DO = doxycycline (30 µg); ENR = enrofloxacin (5 µg); UB = flumequine (UB, 30 µg); CN = gentamicin (10 µg); OXY = oxytetracycline (30 µg); SXT = sulfamethoxazole-trimethoprim (25 µg).

**Table 2:** Antibiotic resistance rates within each of the main six bacterial families isolated from the clinical samples and organs of 60 loggerhead sea turtles.

Bacterial Family	Percentage of Resistant Strains						
	CAZ	DO	ENR	UB	CN	OXY	SXT
*Vibrionaceae*	8.1%	7%	5.8%	1.2%	10.5%	7%	8.2%
*Shewanellaceae*	6.3%	18.8%	6.3%	6.7%	6.3%	6.3%	6.3%
*Pseudomonadaceae*	50%	50%	50%	33.3%	33.3%	50%	66.7%
*Enterobacteriaceae*	60%	60%	60%	40%	60%	40%	40%
*Morganellaceae*	20%	80%	80%	80%	60%	80%	60%
*Enterococcaceae*	75%	0%	20%	60%	100%	0%	0%

CAZ = ceftazidime (30 µg); DO = doxycycline (30 µg); ENR = enrofloxacin (5 µg); UB = flumequine (UB, 30 µg); CN = gentamicin (10 µg); OXY = oxytetracycline (30 µg); SXT = sulfamethoxazole-trimethoprim (25 µg).

## Data Availability

The data presented in this study are available within the article.

## Supplementary Materials

The following supporting information can be downloaded at: https://www.mdpi.com/article/10.3390/ani14142103/s1, Table S1: Hospitalisation information of 60 loggerhead sea turtles admitted at Turtle Point between 2015 and 2023 and subjected to bacteriological analysis; Table S2: Species identification within each of the main six bacterial families isolated from the clinical samples and organs of 60 loggerhead sea turtles.
