# Supplementary material for: Antibiotic Resistance of Bacteria Isolated from Clinical Samples and Organs of Rescued Loggerhead Sea Turtles (Caretta caretta) in Southern Italy
Source: Animals (Basel). 2024 Jul 18;14(14):2103. doi: 10.3390/ani14142103 (PMC11273476; doi:10.3390/ani14142103)
Supplement: Supplementary file 1 [file animals-14-02103-s001.zip › Table S1.pdf]

Table S1. Hospitalization information of 60 loggerhead sea turtles admitted at the Turtle Point between 2015 and 2023 and subjected to bacteriological analysis.

SZN Code	Status	Sex	Weight (kg)	SCL (cm)	CCL (cm)	SCW (cm)	CCW (cm)	Region	Rescue Mode	Arrival Date	Main Clinical Finding	Samples	Strain number	AB Treatment (Yes/No)
V23/15	dead	F	54	ND	73,5	ND	67,1	Other	Stranded	27/07/15	Debilitation	Intestine, Spleen	1	Yes
V24/16	dead	M	15,98	45,9	51,4	40,6	48	Campania	Adrift	19/08/16	Fishing line	Intestine	1	No
V02/17	dead	F	29,35	57,5	63	49,8	61	Campania	Fishermen	09/02/17	Hook and Fishing line	Liver, Intestine, Spleen, Muscle, Lung, Kidney	2	Yes
V21/17	dead	M	68,95	75,3	84	61,7	86,5	Campania	Adrift	14/08/17	Multiple traumas	Spleen	1	Yes
V10/17	alive	ND	29,6	58,6	65,5	49,3	61,5	Campania	Stranded	07/05/17	Fistula	Fistula swabs, Necrotic tissue	4	Yes
V06/18	alive	ND	8,5	38,7	42	34	40,5	Campania	Stranded	02/04/18	Entanglement	Flipper wound swab	4	No
V09/18	dead	F	2,9	27,7	31,2	24	28,8	Campania	Stranded	09/05/18	Debilitation	Liver, Intestine, Lung	3	No
V11/18	alive	ND	6,3	37,4	43,1	31,9	39,5	Campania	Adrift	19/05/18	Fishing line	Coelomic fluid	1	No
V13/18	alive	ND	5,8	34,4	38	28,4	35,5	Other	Adrift	29/05/18	Hook and Fishing line	Coelomic fluid	1	Yes
V03/19	alive	ND	15,1	47	51	38,8	47	Campania	Adrift	17/04/19	Entanglement	Coelomic fluid	1	No
V09/19	alive	F	35,3	67	73,2	53,2	68,5	Campania	Adrift	19/08/19	Ocular lesions	Conjunctival swab	4	Yes
V13/19	alive	F	45,7	66	72,5	53,6	67	Other	Adrift	24/09/19	Debilitation	Oropharynx swab	4	Yes
V14/19	alive	ND	20,45	53	57,5	42,5	52,4	Campania	Adrift	06/11/19	Entanglement	Flipper wound swab	2	No
V15/19	alive	ND	19,9	52,4	57,9	44,4	53,5	Other	Stranded	21/11/19	Entanglement	Flipper wound swab	2	No
V16/19	alive	ND	18,9	53,2	58	44	52,5	Other	Stranded	21/11/19	Entanglement	Flipper wound swab	4	No
V01/20	alive	ND	27,5	57,9	63,5	47,9	60	Other	Stranded	09/01/20	Propeller traumas	Flipper wound swab	1	No
V02/20	alive	ND	0,169	10,4	ND	7,88	ND	Other	Stranded	06/03/20	Cold-stunning	Oropharynx swab	3	No
V06/20	alive	M	54	68	75	54,8	70,5	Campania	Adrift	21/06/20	Propeller traumas	Carapace fracture swab	3	No
V09/20	alive	F	58,45	71,8	78	58,1	74	Campania	Adrift	04/07/20	Buoyancy disorder	Oropharynx swab, Cloaca swab	1	No
V07/20	alive	ND	21,6	53,2	60,5	46	54,8	Other	Adrift	23/06/20	Entanglement	Flipper wound swab	1	Yes
V10/20	alive	ND	25,65	56	62	45,7	55	Other	Adrift	14/07/20	Propeller traumas	Carapace fracture swab	1	No
V12/20	alive	ND	13,55	42,2	48,5	35,3	43	Other	Adrift	20/07/20	Propeller traumas	Carapace fracture swab	1	No
V14/20	alive	ND	24,05	54,5	61	45,4	56	Campania	Adrift	03/08/20	Ocular lesions	Conjunctival swab	2	No
V16/20	alive	ND	17,35	51,8	58	41,3	52,2	Other	Adrift	08/09/20	Entanglement	Flipper wound swab	2	No
V10/21	alive	ND	4,65	33,2	37	28	33	Campania	Adrift	06/02/21	Entanglement	Flipper wound swab	3	Yes
SZN 21/11	alive	ND	6,6	37,6	41,3	32	38,7	Other	Stranded	13/02/21	Entanglement	Lymphatic fluid	2	Yes

SZN 21/18	alive	F	29,5	65,7	71	53,4	66	Other	Adrift	30/03/21	Hook and Fishing line	Coelomic fluid	2	No
SZN 21/19	alive	ND	0,175	10,3	ND	9,1	ND	Other	Adrift	27/04/21	Debilitation	Oropharynx swab, Cloaca swab	4	Yes
SZN 21/23	dead	M	10,7	43,5	49,9	36,9	45,5	Other	Adrift	21/05/21	Pneumocoeloma	Liver	1	No
SZN 21/24	alive	F	49,8	65,2	71,7	50,4	63	Campania	Adrift	22/05/21	Debilitation	Oropharynx swab	2	No
SZN 21/25	alive	F	42,05	63,2	68,5	51,5	64,6	Campania	Adrift	22/05/21	Debilitation	Oropharynx swab	1	No
SZN 21/26	alive	M	47	66,2	73	54	68,3	Campania	Adrift	30/05/21	Buoyancy disorder	Oropharynx swab	3	No
SZN 21/27	dead	F	4,1	34,2	38,3	27,8	34,1	Campania	Adrift	12/06/21	Fishing line	Intestine, Kidney	1	Yes
SZN 21/31	alive	F	32,85	63	70,5	50,9	64	Other	Adrift	25/06/21	Entanglement	Oropharynx swab	1	Yes
SZN 21/30	alive	ND	25,8	56,6	62	46,1	54	Other	Adrift	25/06/21	Entanglement	Oropharynx swab	3	No
SZN 21/35	dead	M	63,9	76	80,2	60,2	75	Campania	Stranded	08/07/21	Multiple traumas	Brain, Intestine, Lung	3	No
SZN 21/37	alive	F	39,8	68	71,2	52	66	Campania	Fishermen	19/07/21	Sea water aspiration	Oropharynx swab, Glottis swab	4	No
SZN 21/45	alive	F	31,55	59,7	65,5	47,8	59	Other	Adrift	28/09/21	Sea water aspiration	Oropharynx swab, Glottis swab	3	Yes
SZN 21/46	alive	ND	11,6	43,6	48	36,5	44,5	Other	Fishermen	28/09/21	Entanglement	Flipper wound swab, Necrotic tissue	3	No
SZN 21/48	alive	ND	0,053	6,55	7,8	5,75	7,5	Other	Stranded	30/12/21	Cold-stunning	Skin swab	3	No
SZN 22/01	alive	ND	3	26,7	30,4	21,9	27,7	Campania	Stranded	22/02/22	Sea water aspiration	Oropharynx swab, Glottis swab	2	No
SZN 22/02	alive	M	50,6	69	78	55,1	70,5	Campania	Fishermen	04/04/22	By-catch	Oropharynx swab	5	No
SZN 21/40	alive	ND	9,75	40,7	45	34	42,5	Other	Stranded	02/08/21	Fishing line	Coelomic fluid	1	Yes
SZN 22/03	dead	F	49	67,5	74,2	54,2	69,8	Campania	Stranded	30/04/22	Multiple traumas	Intestine	1	No
SZN 22/05	alive	ND	3,8	32,5	37	27,3	33	Campania	Adrift	20/05/22	Hook and Fishing line	Coelomic fluid	2	No
SZN 22/06	alive	ND	21,65	52,1	57,8	45,2	55	Other	Adrift	24/05/22	Ocular lesions	Oropharynx swab, Conjunctival swab, Necrotic tissue	3	No
SZN 22/08	alive	ND	15	48	53	38,4	47,5	Other	Adrift	30/08/22	Multiple traumas	Flipper wound swab, Carapace fracture swab	3	No
SZN 22/11	dead	M	3,8	27,5	31	23,7	28	Other	Fishermen	28/10/22	Hook and Fishing line	Intestine, Lung, Kidney	1	Yes
SZN 22/12	alive	ND	7,05	34	38,3	28,2	34	Other	Stranded	16/12/22	Entanglement	Flipper wound swab, Oropharynx swab	4	No
SZN 23/03	alive	ND	21,5	54,5	60	45,2	56	Other	Stranded	23/01/23	Entanglement	Flipper wound swab	2	Yes
SZN 22/07	alive	ND	1,63	19,2	22,5	16,4	20,8	Campania	Adrift	02/06/22	Ocular lesions	Conjunctival swab	1	Yes
SZN 23/01	alive	ND	4,3	30,9	34,3	25,6	30,7	Campania	Adrift	02/01/23	Fishing line	Coelomic fluid	1	No
SZN 23/02	alive	M	41	67,6	75	53,8	67	Campania	Adrift	03/01/23	Entanglement	Skin swab	1	No
SZN 22/04	alive	ND	1,8	19,7	23	17,1	22	Campania	Adrift	09/05/22	Ocular lesions	Conjunctival swab	1	Yes
SZN 23/05	alive	ND	0,098	8,03	9,8	7,02	9,1	Other	Stranded	14/03/23	Buoyancy disorder	Cloaca swab	1	No

SZN 23/07	alive	ND	13,7	45,4	50,7	38,4	47	Other	Adrift	28/03/23	Entanglement	Flipper wound swab	2	No
SZN 23/11	alive	ND	8,75	41	47,4	33,3	40,5	Campania	Fishermen	20/05/23	Multiple traumas	Carapace fracture swab	4	No
SZN 23/12	alive	ND	21,35	50,4	55,5	42,3	51	Campania	Fishermen	12/06/23	Multiple traumas	Carapace fracture swab, Abscess	4	No
SZN 23/13	alive	F	43,3	64,6	70,3	54,3	67,7	Campania	Adrift	11/07/23	Hook	Necrotic tissue	5	No
SZN 23/20	alive	F	29,15	54,7	60	47,5	58	Other	Stranded	06/11/23	Entanglement	Flipper wound swab, Necrotic tissue	5	Yes
