# Supplementary material for: Antibiotic Resistance of Bacteria Isolated from Clinical Samples and Organs of Rescued Loggerhead Sea Turtles (Caretta caretta) in Southern Italy
Source: Animals (Basel). 2024 Jul 18;14(14):2103. doi: 10.3390/ani14142103 (PMC11273476; doi:10.3390/ani14142103)
Supplement: Supplementary file 1 [file animals-14-02103-s001.zip › Table S2.pdf]

Table S2. Species identification within each of the main six bacterial families isolated from clinical samples and organs of 60 loggerhead sea turtles.

Families	Species	Number of strains
Vibrionaceae	*Vibrio alginolyticus*	26
	Vibrio harveyi	22
	Vibrio fluvialis	5
	Vibrio navarrensis	5
	Vibrio diazotrophicus	3
	Vibrio anguillarum	1
	Vibrio furnissii	1
	Vibrio gallicus	1
	Vibrio mediterranei	1
	Vibrio orientalis	1
	Vibrio parahaemolyticus	1
	Vibrio pelagius	1
	Vibrio pelagius II	1
	Vibrio scophthalmi	1
	Vibrio splendidus I	1
	Vibrio splendidus II	1
	Vibrio tubiashii	1
	Vibrio xuii	1
	Photobacterium damselae subsp. *damselae*	4
	Photobacterium swingsii	1
	Grimontia hollisae	1
	*Vibrio spp.**	4
	*Photobacterium spp.**	1
	*Aliivibrio spp.**	1
Shewanellaceae	*Shewanella algae*	13
	Shewanella putrefaciens	3
Pseudomonadaceae	*Pseudomonas aeruginosa*	2
	Pseudomonas putida	2
	Pseudomonas fluorescens	1
	Pseudomonas mendocina	1
Enterobacteriaceae	*Citrobacter freundii*	4
	Enterobacter cancerogenus	1
Morganellaceae	*Morganella morganii* subsp. *morganii*	5
Enterococcaceae	*Enterococcus faecalis*	3
	Enterococcus gallinarum	1
	Vagococcus fluvialis	1
Streptococcaceae	*Lactococcus garviae*	2
Staphylococcaceae	*Staphylococcus sciuri*	1

*These strains were not successfully identified to the species level, and reported at genus level.
